# A Practical Treatise on Diseases of the Kidneys

**Published:** 1885-12

**Authors:** 


*A Practical Treatise on Diseases of the Kidneys.* By C. H. RALFE. Pp. 572. London: H. K. LEWIS. 1885.

We have formed a high opinion of this work. For the ordinary purposes of practitioner and student, we consider it about the best we possess. Perhaps more facts and more philosophy may be found in such works as those of Roberts and Dickinson, but we doubt if a more accurate concrete knowledge of Diseases of the Kidneys could be acquired from these large works than from this small one. That Dr. Ralfe shows a full and perfect acquaintance with his subject, practical as well as bibliographical, is abundantly evident. The arrangement of the work is enough to show this. It is easy enough to write continuously and discursively our individual thoughts and observations on disease: if our thinking is sound, and our observation is exact, our writings may be suggestive and instructive. But, to be of real educational value to the ignorant student, a written work must be arranged according to scientific methods--specific, generic, etiological, and practical. In this sense, Dr. Ralfe's work is a genuine success. Beyond this, criticism need not go. On special points, studied by special men, it is just possible that our author may not give full satisfaction. It is clear, however, that on broad principles he has produced, within reasonable compass, a monograph which, for accuracy and extent of information conveyed, it would be difficult to rival.

